# Si Characterization on Thinning and Singulation Processes for 2.5/3D HBM Package Integration

**DOI:** 10.3390/ma17225529

**Published:** 2024-11-13

**Authors:** MiKyeong Choi, SeaHwan Kim, TaeJoon Noh, DongGil Kang, SeungBoo Jung

**Affiliations:** School of Advanced Materials Science & Engineering, Sungkyunkwan University, 2066 Seobu-ro, Jangan-gu, Suwon 16419, Republic of Korea; byclh@naver.com (M.C.); shboy2029@g.skku.edu (S.K.); hokujoon@skku.edu (T.N.); jeanne9910@skku.edu (D.K.)

**Keywords:** thinning, singulation, FWMH, Si wafer, Raman analysis, 2.5D/3D integration, semiconductor package

## Abstract

As stacking technologies, such as 2.5D and 3D packages, continue to accelerate in advanced semiconductor components, the singulation and thinning of Si wafers are becoming increasingly critical. Despite their importance in producing thinner and more reliable Si chips, achieving high reliability remains a challenge, and comprehensive research on the effects of these processing techniques on Si chip integrity is lacking. In this study, the impacts of wafer thinning and singulation on the fracture strength of Si wafers were systematically compared. Three different grinding processes, namely fine grinding, poly-grinding, and polishing, were used for thinning, and the resulting surface morphology and roughness were analyzed using scanning electron microscopy and an interferometer. In addition, the residual mechanical stress on the wafer surface was measured using Raman spectroscopy. The fracture strength of Si wafers and chips was assessed through three-point bending tests. Singulation, including blade dicing, laser dicing, and stealth dicing, was evaluated for its impact on fracture strength. Among these processes, polishing for wafer thinning exhibited the lowest full-width half maximum and intensity ratio of Raman shifts (I480/I520), indicating minimal residual stress and surface defects. Consequently, Si wafers and chips processed through polishing demonstrated the highest fracture strength. Moreover, the 60 µm thick Si wafers and chips showed the highest fracture strength compared with those with thicknesses of 90 and 120 µm, possibly because of the increased flexibility, which mitigates stress. Among the singulation methods, stealth dicing yielded the highest fracture strength, outperforming blade and laser dicing. The combination of wafer thinning via polishing and singulation via stealth dicing presents an optimal solution for producing highly reliable Si chips for 2.5D and 3D packaging. These findings may be valuable in selecting optimal processing technologies for high-reliability Si chip production in industrial settings.

## 1. Introduction

As the density of CPUs, GPUs, and AI chips, which are critical components in high-performance computing and cloud services, continues to increase, system design and architecture for achieving high performance have become increasingly vital. Consequently, advanced packaging technologies for AI and 5G applications are evolving toward 2.5D and 3D package structures or heterogeneous integrated packages that can accommodate the rapidly growing number of input/output (I/O) connections required for faster speeds [1,2].

**Figure 1:**
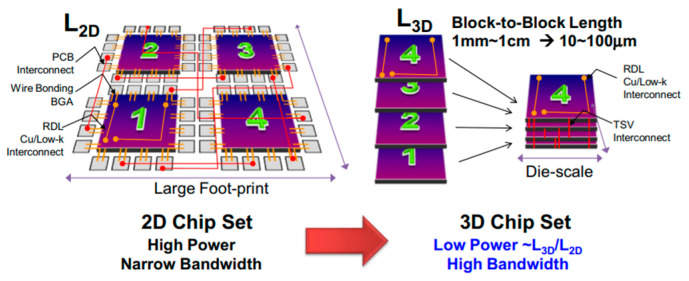
Advantages over 2D chip set for changing from 2D to 3D packages in structure: high bandwidth.

Initially, advanced packaging possesses system-in-package and package-on-package (PoP) structures. However, at present, advanced packaging has evolved into 3D stacked package structures using a through-silicon via (TSV) technology to meet stringent design rules and thin form factor requirements [3,4,5]. TSV-based 3D packaging has received considerable attention as a potential solution to address the limitations of conventional 2D scaling and I/O. As shown in Figure 1, 3D packages such as high-bandwidth memory (HBM) have several advantages, including reduced signal loss, minimized connection delays, smaller form factors, and lower power consumption. Recently, considerable attention has been paid to improving productivity and reducing costs in high-volume manufacturing [6,7,8,9,10].

Traditionally, flip-chip ball grid array packages have been widely adopted for advanced electronic packaging because of their high electrical performance and ability to support numerous I/O connections. However, the industry has shifted from 2D to 2.5D and 3D package structures to enhance the functionality and performance of semiconductor packages. This transition has led to thinner Si chips, the increasing use of organic and Si interposers, and the need for optimized wafer thinning and singulation, both of which are essential for 3D packaging [11,12,13]. As 3D packaging technologies continue to evolve, wafer thinning and singulation have re-emerged as critical technologies for ensuring the performance and reliability of advanced semiconductor packages. The ongoing development of 3D stack packaging has resulted in thinner Si chips, which reduce the overall package height, thereby promoting higher performance and smaller form factors. Thinner Si chips allow for higher design flexibility, lower electrical resistance, and enhanced signal characteristics within the stacked package [14]. In addition, thinner wafers provide improved heat dissipation characteristics because of the decreased thermal resistance, with thicknesses being reduced to as low as 20 µm [15]. In addition, there are some famous studies about the electronic devices on Si substrates: thermoelectric device, memory, etc. In the case of thermoelectric device application, Si nanodots and Si films containing epitaxially grown Ge nanodots were introduced with reduced thermal conductivity and the conservation of high electrical conductivity as Si-based thermoelectric materials [16]. At the other representative memory application, the doping amount of a silicon substrate can provide for different synaptic functions, which include long-term memory and short-term memory in a TiO_2_-based resistive memory device with a silicon bottom electrode [17].

In general, Si wafer thinning involves steps such as grinding, etching, and polishing, each requiring precision and uniformity. Wafer singulation, which separates individual Si chips from the wafer, is generally carried out through dicing or sawing. This process demands high accuracy to prevent damage to the delicate Si chips. Advanced techniques such as laser dicing provide higher precision and lower stress compared with traditional mechanical dicing, and they have received considerable attention in recent years.

Considering that silicon is inherently a hard and brittle material, mechanical grinding is commonly used to achieve the target wafer thickness. Most grinding systems employ a two- or three-step process, starting with coarse grinding to quickly remove up to 80% of the wafer, followed by fine grinding to reduce surface roughness and minimize damage to the wafer. According to Chen et al. [18], the back-grinding of Si wafers can introduce defects such as microcracks and amorphous regions on the wafer surface. Although single-crystal silicon is inherently stable, these defects reduce its fracture strength. However, McLellan et al. reported that the fracture strength of Si wafers increases as the surface quality improves following thinning [19].

Despite these improvements, 3D stacked packages such as HBM require thin Si chips, which leads to a higher frequency of microcracks during wafer thinning. Thus, grinding such as micro-grinding, poly-grinding, and polishing must be carefully selected and applied in sequence to minimize residual stress and surface defects, thereby enhancing the fracture strength of Si chips.

Furthermore, Si wafers are singulated into smaller Si chips. Thus, their fracture strength may decrease further during this process. Therefore, understanding the effects of various singulation techniques on the fracture strength of Si chips is necessary to minimize any potential degradation [20,21]. In recent years, wafer thinning and singulation have become the critical manufacturing steps in advanced HBM, PoP, 2.5D, high density TSV (through-silicon vias) and 3D semiconductor packaging [22,23,24]. For example, laser dicing, a widely used singulation method for thin Si wafers, has received attention in the semiconductor industry because of its cost-effectiveness, faster cutting speed, reduced damage, and narrower kerf width compared with traditional blade dicing [25].

In this study, we compared the fracture strength of Si wafers with three different thicknesses (60, 90, and 120 µm) processed using the following three different grinding methods: fine grinding, poly-grinding, and polishing. In addition, the fracture strength of Si chips singulated from these wafers by blade dicing, laser grooving, and stealth dicing was evaluated. The surface morphology was examined using scanning electron microscopy (SEM), whereas the surface roughness was measured using an interferometer. Local stress residues were characterized using micro-Raman spectroscopy. Finally, the fracture strength was assessed through three-point bending tests.

This study provides valuable insights into the effects of wafer thinning and singulation on the fracture strength of Si wafers and Si chips. Our findings, including analysis of surface roughness, Raman spectroscopy (I480/I520 ratio and full-width half maximum (FWHM)), and fracture mechanisms, provide important implications for optimizing these processes to enhance the reliability of advanced semiconductor packages.

## 2. Materials and Methods

A 12-inch dummy wafer (780 µm thick Si wafer without circuit) with a (1 1 1) orientation was used in this study. Three wafer thicknesses were prepared using different grinding processes (Table 1): fine grinding, poly-grinding, and polishing. During coarse grinding, wheels with grit sizes of 1000 were used. During fine grinding and poly-grinding, wheels with grit sizes of 5000 and 8000 were used. Polishing, which involved chemical treatment, used an SiO_2_ slurry. The grinding conditions for wafer thinning to different target thicknesses (60, 90, and 120 µm) are summarized in Table 1.

After wafer thinning, the Si wafers were diced into 4 mm × 8mm chips. For rough grinding, a wheel with a grit size of 1000 was used. The singulation conditions for the Si wafers of various thicknesses (60, 90, and 120 µm) are detailed in Table 2. These wafers were cut using three different singulation processes: blade dicing, laser grooving, and stealth dicing After wafer thinning, the surface topography was analyzed using field-emission SEM. Surface roughness was measured using an interferometer (Interferometry, Bruker, Karlsruhe, Germany). Interferometry was performed in a 0.5 μm × 0.5 µm scanning area for surface analysis. Residual stress in the wafers was analyzed by Raman spectroscopy (Lab Ram Aramis, Horiba Jobin Yvon, Palaiseau, France), which provides insights into the defect levels and residual stress within the crystal lattice, as these factors affect the optoelectronic properties of the material. The peak positions and FWHM obtained from Raman spectroscopy were used to evaluate the residual stress levels in wafers processed using different thinning methods [26,27,28].

The fracture strength of the wafers and Si chips was also evaluated on the basis of the residual stresses introduced by the three thinning processes and singulation methods. The fracture strength was measured using a three-point bending test (Instron, Chicago, IL, USA) for wafers and chips of varying thicknesses (60, 90, and 120 µm).

In this study, the feasibility of different singulation and grinding methods commonly used in wafer and chip processing was explored to determine their impact on fracture strength and overall wafer integrity.

## 3. Results

**Figure 2:**
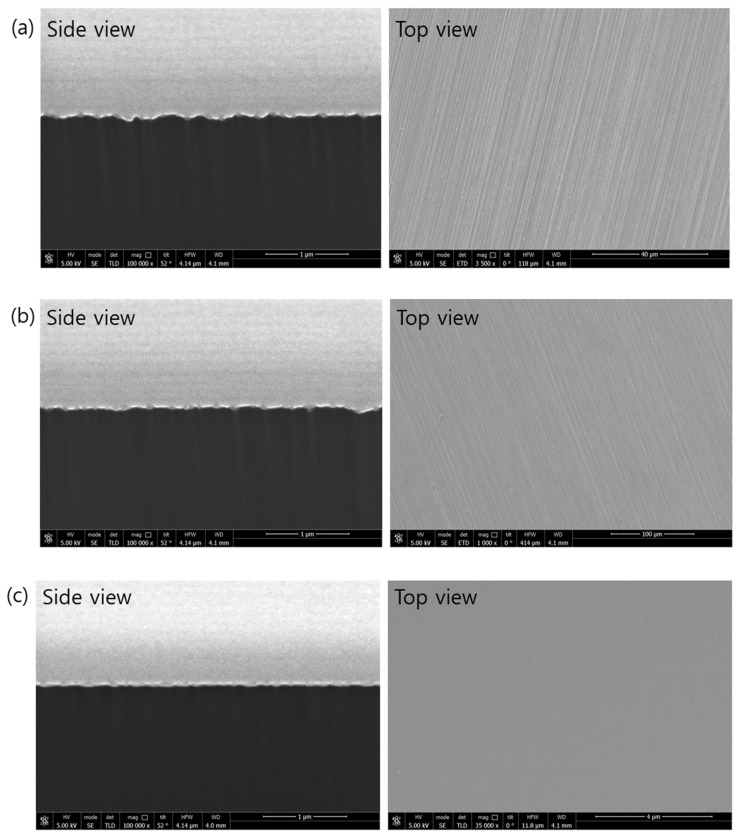
Cross-sectional SEM micrographs and the surface of wafer with different grinding processes: (**a**) fine grinding, (**b**) poly-grinding, and (**c**) polishing.

Figure 2 presents the cross-sectional SEM micrographs and surface images of 120 µm thick silicon chips processed using different grinding techniques. Among the processes, polishing produced the smoothest surface morphology (Figure 2).

**Figure 3:**
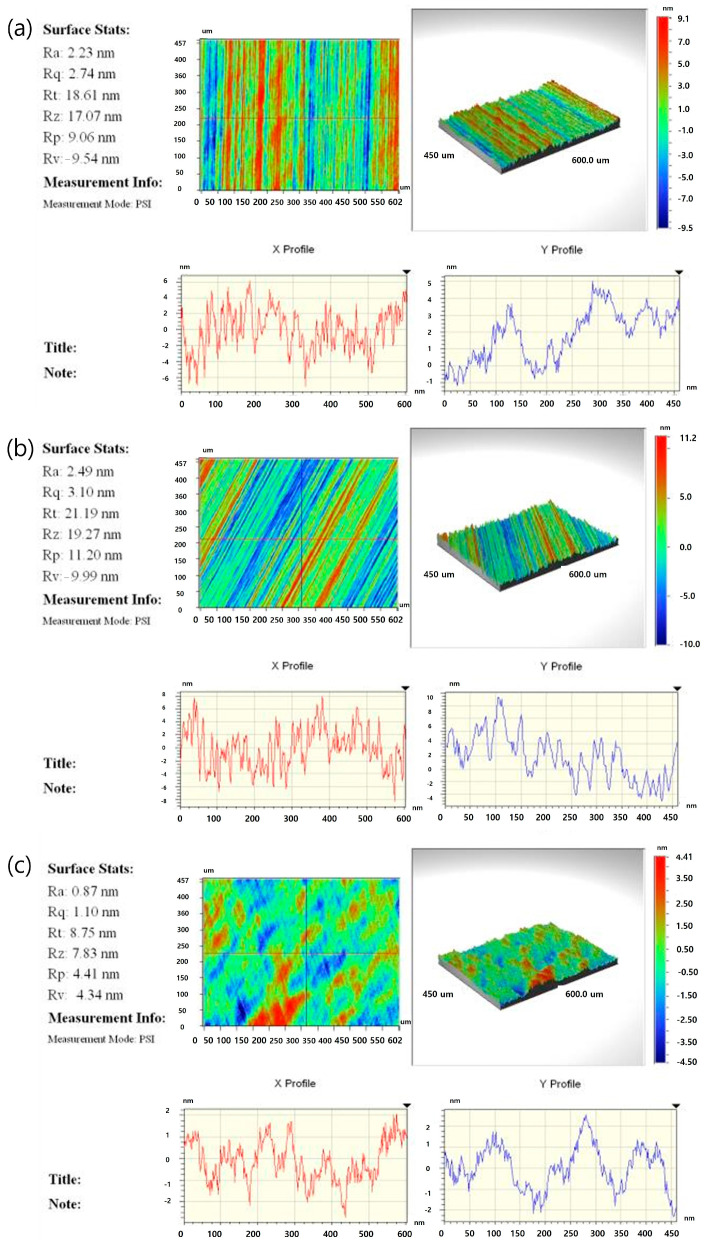
Roughness and micrographs on the surface of Si chips for grinding conditions on the same wafer with a thickness of 120 μm: (**a**) fine grinding, (**b**) poly-grinding, and (**c**) polishing.

Figure 3a–c depicts the surface roughness of 120 µm thick wafers processed by fine grinding, poly-grinding, and polishing. Polishing yielded the lowest surface roughness compared with the other two methods (Figure 3a–c). By contrast, the surfaces of wafers subjected to fine and poly-grinding exhibited clearly visible grinding marks, which could serve as initiation points for wafer cracks and propagate easily. The X and Y depth profiles of the silicon wafer surface further reveal the roughness and morphology of the silicon (1 1 1) crystal orientation [29]. According to Zarudi and Zhang [30], the grinding of thin Si wafers can introduce or exacerbate crystallographic defects such as dislocations and stacking faults. These defects remarkably reduce the fracture strength of wafers processed by different thinning methods, emphasizing the importance of optimizing grinding conditions for Si wafers and chips. These defects may become more pronounced under thermal stress, particularly during annealing at 450 °C and above [29,31]. Jiun et al. [32] also reported that the local stresses induced by these defects differ from those in a perfect silicon crystal lattice. Furthermore, such stress can be alleviated by using specialized polishing techniques involving chemical treatments, which effectively reduce defect-induced stress. Considering that interferometer provides superior analytical resolution compared with phase shifting, the surface roughness of wafers processed using different grinding techniques was analyzed in detail across the edge and center regions of the wafer.

**Figure 4:**
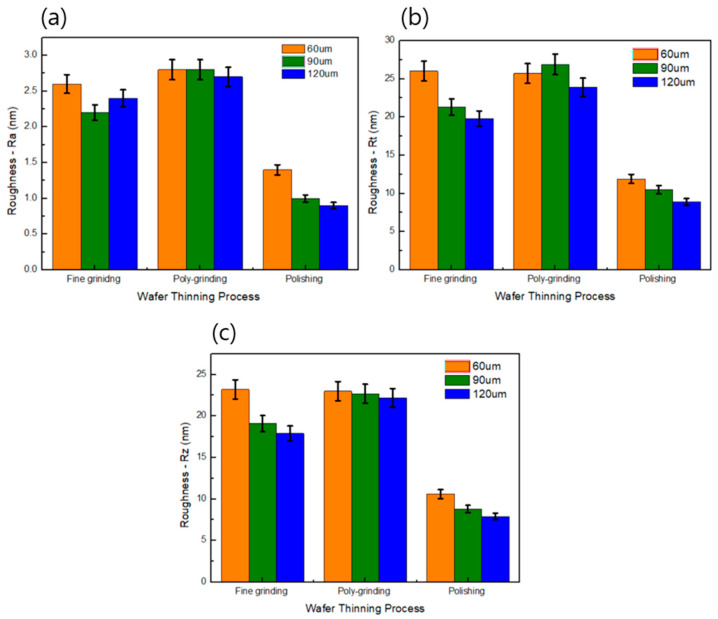
Roughness (**a**) Ra, (**b**) Rt, and (**c**) Rz of Si wafers ground by different grinding processes at a thickness of 60 µm, 90 µm, and 120 µm.

The roughness of Si wafers processed using different grinding processes was consistent across samples (Figure 4a–c). The measurements for roughness of Si wafers were recorded in terms of Ra, Rt, Rz, as follows:

- Ra: Arithmetic average of the absolute values of the surface height deviations measured from the mean plane;
- Rt: Maximum vertical distance between the highest and lowest data points in the image following the plane fit;
- Rz: Average difference in height between the highest peaks and valleys relative to the mean plane.

Under fine grinding conditions, the Ra value of Si wafer thickness 90 µm was the lowest, the Rt value of Si wafer thickness 120 µm was the lowest, and the Rz value of Si wafer thickness 120 µm was the lowest. The deviation of Ra, Rt, and Rz with poly-grinding were lower than for both fine grinding and polishing with respect to Si thickness variance.

In view of Si wafer thickness variances, Ra, Rt, and Rz of 60 µm Si wafer thickness had almost same values under fine grinding and poly-grinding conditions. Despite using a finer mesh wheel for thinning processes compared to fine grinding, poly-grinding showed a higher roughness value. This suggests that wheel marks may not be effectively removing or die warpage by die shape or die thickness. However, wafers polished using the polishing process exhibited the lowest roughness compared with those processed by fine grinding and poly-grinding. The smoother surface produced by polishing indicated that grinding marks and residual microdefects were effectively removed during the process. However, wafers polished using the polishing process exhibited the lowest roughness compared with those processed by fine grinding and poly-grinding. The smoother surface produced by polishing indicated that grinding marks and residual microdefects were effectively removed during the process.

The mechanical stress induced during wafer thinning was measured using micro-Raman spectroscopy. The Raman signal originates from a volume determined by the laser’s wavelength and beam diameter, with shorter wavelengths providing more detailed information on residual surface stress. The total scattered light intensity from the surface to a specific depth is correlated with stress levels, as proposed by Takahashi and Makino [33]. Raman spectroscopy was used to evaluate the residual stress on the wafer surface using a 514 nm laser wavelength and a 50× magnification objective lens, resulting in a spot size of less than 1 µm and a laser power below 1 mW.

A Raman peak represents a specific vibrational mode in the spectrum obtained from this inelastic scattering, and the position of each peak correlates to the vibrational frequency of the molecule. FWHM refers to the width of a Raman peak measured at half of its maximum height. The FWHM of the Raman peak shows material defects on the quality of the thinning Si wafer. An increase in defects generally leads to broader peaks, which means phonon’s life reduction caused by lattice vibration of Si cannot fully transfer to total crystal.

**Figure 5:**
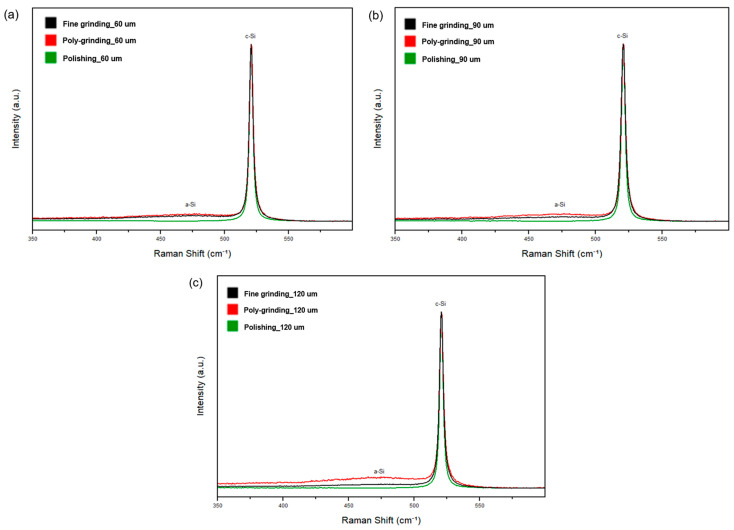
Raman peaks of wafers thinned by using three grinding processes: (**a**) 60 µm, (**b**) 90 µm, and (**c**) 120 µm.

Figure 5a–c show the Raman peaks of wafers thinned by different grinding processes at different thickness 60, 90, and 120 µm. The peaks for all three wafers were located around 521 cm⁻¹, corresponding to the characteristic c-silicon peak. The peak shift near 521 cm⁻¹ is particularly important, as it provides insight into the local residual stress on the wafer surface [31,32,34]. Wolf also reported that the Raman peak frequency for silicon decreases near lines of tensile stress [16]. As shown in Figure 5a–c, the polished wafers exhibited the lowest Raman peak shift within the a-silicon range (450 cm⁻¹ to 500 cm⁻¹), indicating the least residual stress compared to wafers processed by fine grinding and poly-grinding. This confirms that wafers thinned by polishing experience lower residual stress than those processed by other methods.

**Figure 6:**
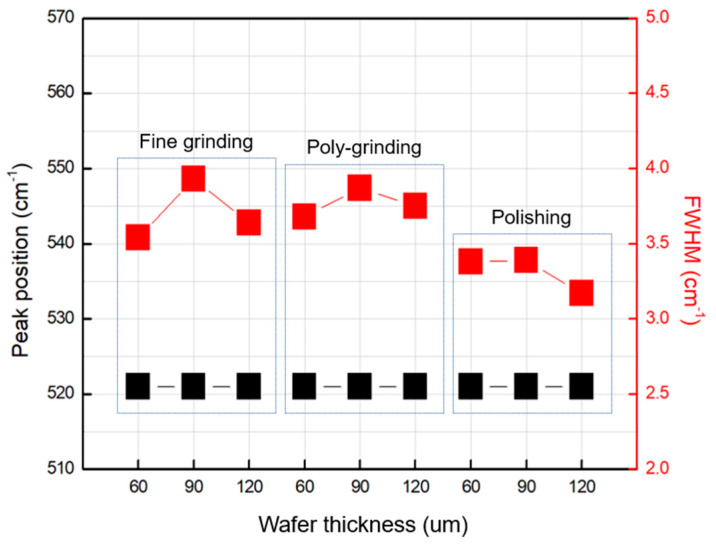
Position of the Raman peak and full-width at half maximum of the wafers thinned using three grinding processes (fine, poly, and polishing) at different thicknesses (60, 90, 120 µm).

Figure 6 displays the FWHM of the c-silicon peaks for wafers processed by fine grinding, poly-grinding, and polishing. The FWHM values for polished wafers were lower than those for wafers subjected to fine grinding and poly-grinding, indicating fewer imperfections and lower residual stress. The combination of lower surface roughness and narrower FWHM suggests that polishing reduces defects more effectively than the other grinding methods.

**Figure 7:**
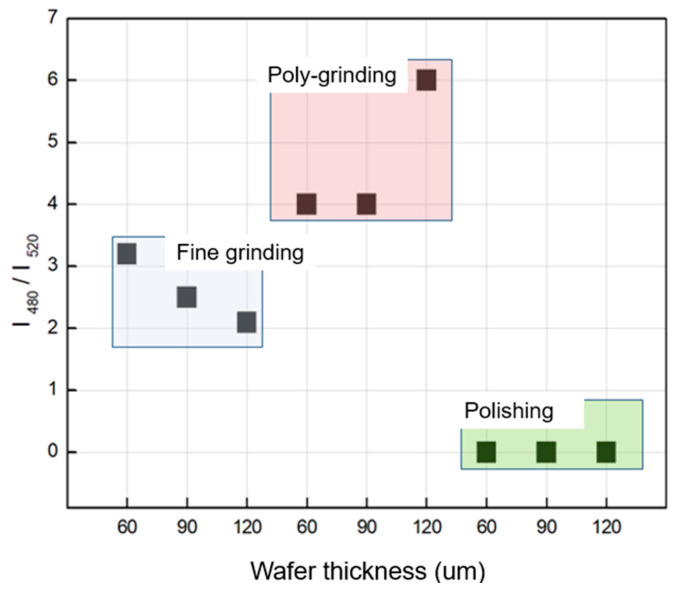
Ratio of intensities integrated at the Raman shift and I480/I520 of the wafers thinned using three grinding processes (fine, poly, and polishing) at different thicknesses (60, 90, and 120 µm).

Figure 7 illustrates the intensity ratio of Raman shifts, I480/I520, for wafers processed by fine grinding, poly-grinding, and polishing. This ratio reflects the size of silicon crystals and their crystalline or amorphous state. As shown in Figure 7, the polished wafers exhibited the lowest I480/I520 ratio, indicating a higher degree of crystallinity and lower levels of amorphous silicon. The low ratio also suggests that the wafer’s surface is smoother, experiences less residual stress, and maintains a higher crystalline state compared to wafers processed by fine grinding or poly-grinding.

**Figure 8:**
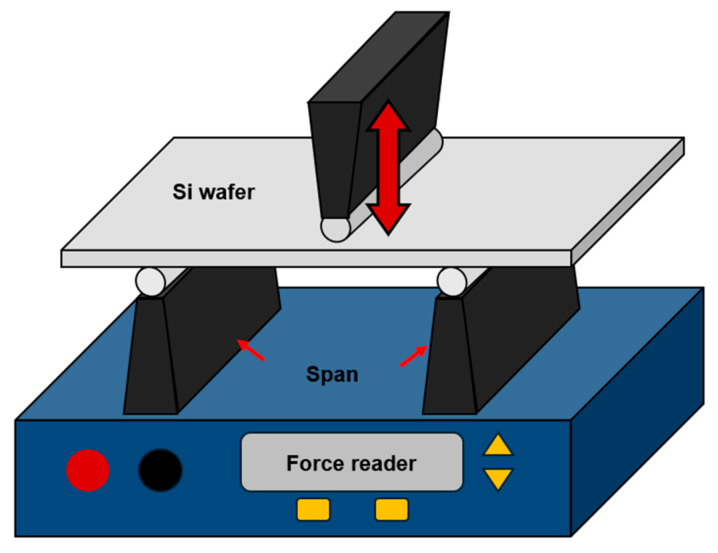
Schematic diagram of the three-point bending test method.

For further investigation of integrity, the fracture strengths of Si wafers and Si chips were evaluated using a three-point bending test with different thinning and singulation processes. The fracture test was conducted at a speed of 1 mm/min with a support span of 2.4 mm, as depicted in Figure 8.

**Figure 9:**
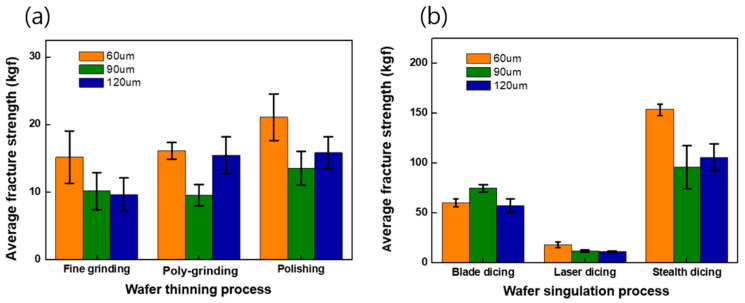
Fracture strength of Si wafer and Si chip with different thinning and singulation processes: (**a**) fracture strength of Si wafer with different thinning processes and (**b**) fracture strength of Si chip with different singulation processes.

Figure 9a shows the fracture strength of wafers processed by different grinding processes. Overall, the wafers thinned by polishing exhibited the highest fracture strength compared to those processed by fine grinding and poly-grinding. For instance, the fracture strength of a 60 µm thick wafer thinned by polishing was measured at 21.2 kgf, higher than wafers processed by fine grinding and poly-grinding at the same thickness. The chemical treatment involved in the polishing process likely contributed to this improvement by reducing surface stress and eliminating surface defects.

Furthermore, the 60 µm thick wafers demonstrated higher fracture strength than the 90 µm and 120 µm thick wafers. This suggests that thinner wafers, particularly those processed by polishing, are more flexible and better able to relieve stress, leading to higher fracture strength. The lower FWHM and I480/I520 ratios in polished wafers further support these findings.

Figure 9b also presents the fracture strength of Si chips produced by three singulation processes: blade dicing, laser grooving, and stealth dicing. The fracture strength of 60 µm thick Si chips produced by stealth dicing was 153 kgf, the highest among all singulation methods. Additionally, 60 µm thick chips consistently exhibited a higher fracture strength compared to 90 µm and 120 µm thick chips within the same singulation process. The increased fracture strength of chips processed by stealth dicing can be attributed to reduced stress concentration at the chip edges and the increased flexibility of thinner chips, which better accommodate stress and reduce the risk of crack propagation.

## 4. Conclusions

Wafer and Si chip thinning and singulation are critical processes in semiconductor manufacturing. As HBM in 3D packages is becoming more important, the demand for thinner chips is growing. However, various thinning processes remarkably affect the mechanical strength of wafers and Si chips. In this study, the effects of three different thinning and singulation processes on the fracture strength of Si wafers and Si chips were investigated.

Surface roughness measurements were conducted using an interferometer. The results indicated that the surface roughness decreased in the following order: poly-grinding, fine grinding, and polishing. Raman spectroscopy was used to measure the residual stress and crystal structure of wafers processed by using different methods, with smoother surfaces corresponding to lower FWHM and I480/I520 ratios. Wafers polished through the polishing process exhibited the lowest residual stress levels.

With regard to the singulation methods, Si chips singulated using stealth dicing demonstrated the lowest FWHM and I480/I520 ratios. Consequently, Si chips processed by stealth dicing exhibited the highest fracture strength compared with chips processed by blade dicing and laser dicing.

Polishing remarkably alleviated residual stress compared with fine grinding and poly-grinding. The combination of polishing and stealth dicing resulted in the lowest mechanical stress levels after thinning and singulation. In addition, such a combination effectively reduced stress concentrations, thereby enhancing the fracture strength of silicon chips. These findings are crucial for ensuring the mechanical reliability of silicon chips in advanced 2.5D and 3D packaging applications.

## Figures and Tables

**Table 1:** Matrix of experiments for wafer thinning.

		Initial Wafer Thickness (µm)	Thickness After Coarse Grinding (µm)	Thickness After Fine Grinding (µm)	Thickness After Fine Grinding (µm)	Thickness After Chemical Treatment
Sample	Fine grinding	780	500	60, 90, 120	-	-
	Poly-grinding	780	500	-	60, 90, 120	60, 90, 120
	Polishing	780	500	-	-	-
Grinding Mesh		-	Mesh 1000	Mesh 5000	Mesh 8000	Mesh 8000 SiO_2_ slurry
Grinding Speed (RPM)		-	1700	2500	2500	250
Feed Rate (µm/s)		-	0.1~2	0.2~1	0.2~1	0.2~0.4

**Table 2:** Conditions of wafer singulation.

Wafer Singulation	Method of Wafer Singulation
Blade dicing	Wafer singulation by cutting blade
Laser grooving	Grooving creation by laser irradiation and follow wafer singulation by cutting a blade
Stealth dicing	Laser irradiation on wafer backside and follow wafer singulation by force loading to mount tape

## Data Availability

The original contributions presented in this study are included in the article. Further inquiries can be directed to the corresponding author.
